# Meta-Analysis of Spatial Navigation Skills in Mild Cognitive Impairment and Alzheimer’s Disease

**DOI:** 10.1093/geroni/igaf122.3811

**Published:** 2025-12-31

**Authors:** Dorota Kossowska-Kuhn, Gillian Gouveia

**Affiliations:** Florida State University, Tallahassee, Florida, United States; Florida State University, Tallahassee, Florida, United States

## Abstract

Dementia affects millions worldwide, with spatial navigation impairments emerging early and becoming more pronounced as the disease progresses. Spatial navigation, the ability to orient oneself and move effectively through the environment, is essential for independent living, yet its trajectory across the dementia spectrum remains insufficiently characterized. Difficulties in spatial navigation manifest in both individuals with Mild Cognitive Impairment (MCI) and Alzheimer’s Disease (AD). Two previous meta-analyses demonstrated significant differences (p < .001) in navigation skills between cognitively healthy older adults (CHOA) and individuals with MCI, with a large effect size (Hedges’ g = 0.88), and between CHOA and individuals with AD, with a very large effect size (Hedges’ g = 1.80). The present meta-analysis compared spatial navigation performance in individuals with MCI and AD by synthesizing evidence from 21 studies, yielding 57 effect sizes. Results indicated that individuals with AD performed significantly worse on spatial navigation tasks than those with MCI, with a small effect size (Hedges’ g = 0.16, p < .001). These findings are based on a diverse range of spatial navigation tasks administered through real-world, virtual reality, computer-based, and paper-and-pencil formats, and encompass various subtypes of navigation including arena, environmental, maze, map drawing, and matrix tasks. This synthesis highlights the need for further research investigating spatial navigation performance differences across the dementia spectrum. Future studies should prioritize longitudinal designs and standardized task protocols to better map trajectories of decline and improve the clinical utility of spatial navigation assessments.

